# Chronic Mild Stress Modified Epigenetic Mechanisms Leading to Accelerated Senescence and Impaired Cognitive Performance in Mice

**DOI:** 10.3390/ijms21031154

**Published:** 2020-02-10

**Authors:** Dolors Puigoriol-Illamola, Mirna Martínez-Damas, Christian Griñán-Ferré, Mercè Pallàs

**Affiliations:** 1 Pharmacology Section, Department of Pharmacology, Toxicology and Therapeutic Chemistry, Faculty of Pharmacy and Food Sciences. University of Barcelona, Av Joan XXIII 27-31, 08028 Barcelona, Spain; dolo.puigoriol@gmail.com (D.P.-I.); christian.grinan@ub.edu (C.G.-F.); 2 Institute of Neuroscience, University of Barcelona (NeuroUB), Campus Mundet, Edifici de Ponent Passeig de la Vall d’Hebron, 171 08035 Barcelona, Spain; 3 Institute of Biomedical Research (IIBO), Universidad Nacional Autónoma de México (UNAM), Subdirección de Enseñanza e Investigación, División de Investigación Biomédica, Centro Médico Nacional 20 de Noviembre, 04510 Ciudad de México, CDMX, Mexico; damas.87@gmail.com

**Keywords:** stress, epigenetics, senescence, cognition, age-related cognitive decline, Alzheimer’s disease, SAMP8, SAMR1, oxidative stress, inflammation, autophagy

## Abstract

Cognitive and behavioural disturbances are a growing public healthcare issue for the modern society, as stressful lifestyle is becoming more and more common. Besides, several pieces of evidence state that environment is crucial in the development of several diseases as well as compromising healthy aging. Therefore, it is important to study the effects of stress on cognition and its relationship with aging. To address these queries, Chronic Mild Stress (CMS) paradigm was used in the senescence-accelerated mouse prone 8 (SAMP8) and resistant 1 (SAMR1). On one hand, we determined the changes produced in the three main epigenetic marks after 4 weeks of CMS treatment, such as a reduction in histone posttranslational modifications and DNA methylation, and up-regulation or down-regulation of several miRNA involved in different cellular processes in mice. In addition, CMS treatment induced reactive oxygen species (ROS) damage accumulation and loss of antioxidant defence mechanisms, as well as inflammatory signalling activation through NF-κB pathway and astrogliosis markers, like Gfap. Remarkably, CMS altered mTORC1 signalling in both strains, decreasing autophagy only in SAMR1 mice. We found a decrease in glycogen synthase kinase 3 β (GSK-3β) inactivation, hyperphosphorylation of Tau and an increase in sAPPβ protein levels in mice under CMS. Moreover, reduction in the non-amyloidogenic secretase ADAM10 protein levels was found in SAMR1 CMS group. Consequently, detrimental effects on behaviour and cognitive performance were detected in CMS treated mice, affecting mainly SAMR1 mice, promoting a turning to SAMP8 phenotype. In conclusion, CMS is a feasible intervention to understand the influence of stress on epigenetic mechanisms underlying cognition and accelerating senescence.

## 1. Introduction

Aging is a multifactorial process characterized by a progressive loss of physiological integrity, resulting in an impaired function and increased vulnerability to death. Physiological brain aging involves cognitive impairment, which includes decreased learning and memory abilities and slower responses to different stimulus [1]. Indeed, normal aging differs from pathological aging and this might be explained by the lifestyle. Environmental factors drive epigenetic modifications, resulting in phenotypic differences, which alter almost all tissues and organs. Although the brain is one of the most affected structures, such modifications lead to a progressive decline in the cognitive function and create a favourable context for the development of neurodegenerative diseases [2]. Aging is the most significant risk factor for most chronic diseases, such as age-related cognitive decline and Alzheimer’s disease (AD) [3]. In fact, AD is the most prevalent dementia and its progression is influenced by both genetic and environmental factors [4].

Several studies define that a good environment is essential to enhance learning and cognitive abilities, besides the continued presence of stressors being associated with the opposite effects. Epigenetics refers to potentially heritable and environmentally modifiable changes in gene expression mediated via non-DNA encoded mechanisms [5]. Recent evidence has demonstrated significant associations between epigenetic alterations and stress [2,6]. Potential threats cause a course of action releasing numerous transmitters and hormones throughout our body, particularly catecholamines and glucocorticoids. The interaction of glucocorticoids and adrenergic systems in specific brain regions has proved an essential mediating mechanism for a wide variety of actions displayed by stress on cognition [7]. Firstly, stress response allows body adaptation. However, when stress is prolonged it has been shown to decrease synaptic plasticity [8], alter hippocampal volume [9,10] and neurotransmitters [11], and may lead to AD [12]. In particular, chronic stress is associated with increased Aβ deposits and hyperphosphorylated Tau [13].

Oxidative stress (OS) and inflammation are deeply involved in age-related deleterious disorders. Along with the aging process, several factors, such as a naturally decreased capacity of the antioxidant enzymes system, create an imbalance between antioxidant mechanisms and reactive oxygen system (ROS)–production equilibrium, accumulating ROS beyond the detoxifying capacity of the antioxidant system resulting in OS, eventually causing cellular damage that can no longer be repaired by internal mechanisms, and finally causing dysfunction of the system [14]. In addition, one of the major changes during aging is the dysregulation of the immune response, leading to chronic systemic inflammatory state [15]. Overall, OS imbalance, mitochondrial dysfunction and the inflammatory response have been linked to accelerated aging and faster progression of neurodegenerative diseases [14,16]. Moreover, the accumulation of dysfunctional and damaged cellular proteins and organelles occurs during aging, resulting in a disruption of cellular homeostasis and progressive degeneration and increases the risk of cell death [17]. Autophagy is particularly important in the maintenance of homeostasis, as it participates in the elimination of disrupted proteins and molecules and helps to maintain a clean cellular environment. It has been widely described that there are alterations in autophagy during aging as well as in age-related cognitive decline, AD and other dementias [18,19,20]. Moderating all these detrimental components is key in the promotion of cell survival and longevity.

The senescence-accelerated mouse (SAM) models include senescence-accelerate prone (SAMP) and senescence-accelerated resistant (SAMR) mice [21]. SAMP8 strain has been widely used as an aging model for the study of brain aging and age-related pathologies. Its cognitive impairment is associated with alterations in both hippocampal structure and activity, causing oxidative stress imbalance and driving to AD neuropathology, such as tau- and amyloid-related alterations [1,22]. On the contrary, SAMR1 strain manifests a normal aging process and is considered as the control strain for SAMP8.

Understanding the epigenetic modifications that a stressful environment triggers in neurodegeneration mechanisms is essential to develop novel therapeutics for age-related cognitive decline. This study aims to determine the effects on brain function of a stressful lifestyle in an animal model with accelerated senescence, SAMP8, and in the resistant senescence strain, SAMR1.

## 2. Results

### 2.1. Epigenetic Modulation Triggers Compacted Chromatin after Chronic Mild Stress

In attempting to systematically address which are the epigenetic modifications caused by chronic mild stressful stimuli, we studied the major epigenetic marks that include histone modifications, DNA methylation and non-coding RNAs.

**Figure 1:**
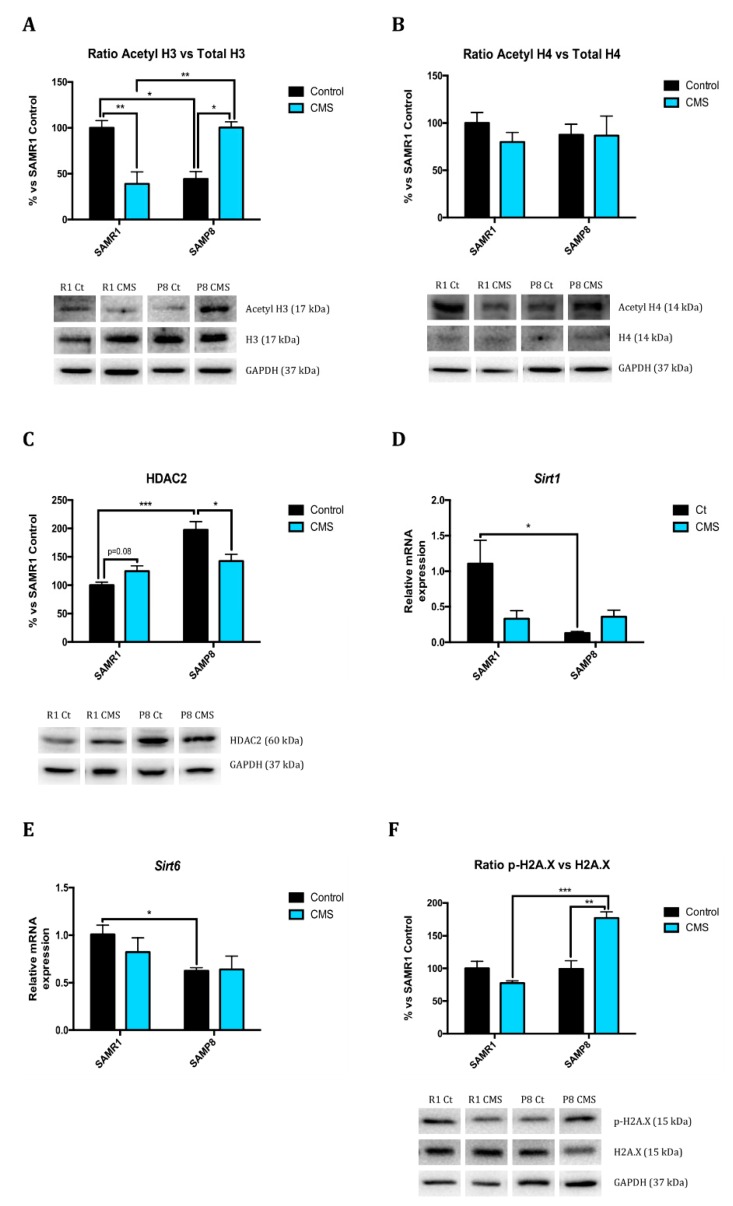

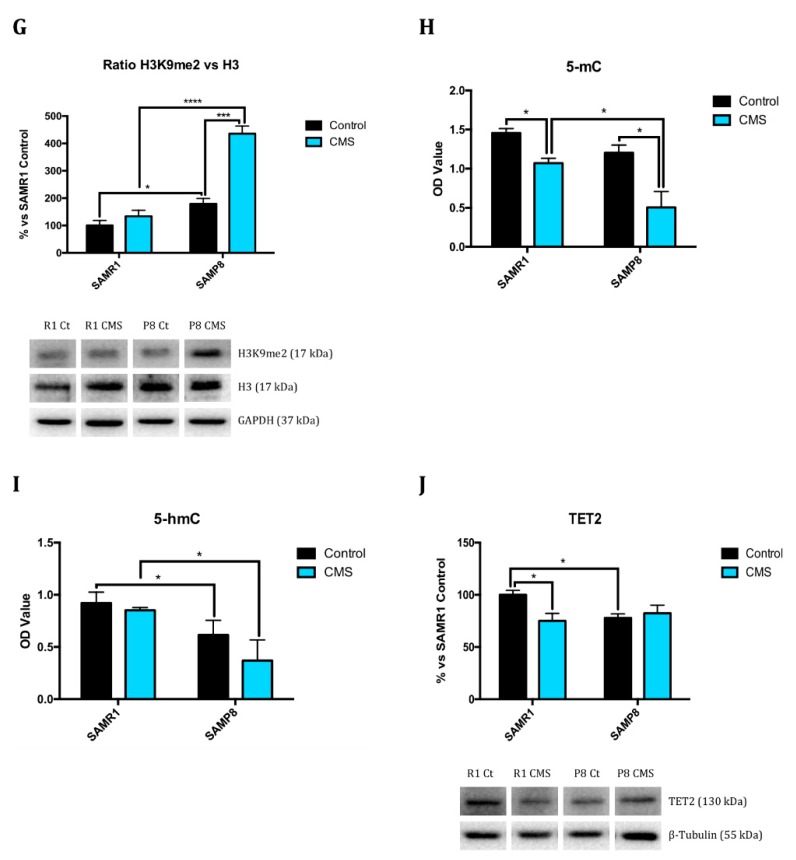
Epigenetic marker changes after CMS. Representative Western Blot for the ratio of Acetylated at Lys9 H3 protein levels and quantification (**A**), the ratio of Acetylated at Lys 12 H4 protein levels and quantification(**B**), HDAC2 protein levels and quantification (**C**), the ratio of p-H2A.X protein levels and quantification (**F**), the ratio of H3K9me2 protein levels and quantification (**G**), and TET2 protein levels and quantification (**J**). Relative gene expression of *Sirt1* (**D**) and *Sirt6* (**E**). Global 5-methylated cytosine (**H**) and 5-hydroxymethylated cytosine levels (**I**). Gene expression levels were determined by real-time PCR. Values in bar graphs are adjusted to 100% for protein levels of SAMR1 control (R1 Ct). Values are mean ± Standard error of the mean (SEM); (*n* = 4 for each group). * *p* < 0.05; ** *p* < 0.01; *** *p* < 0.001; **** *p* < 0.0001.

Regarding histone modifications, we studied global histone 3 (H3) and histone 4 (H4) acetylation, phosphorylation of histone 2A (H2A.X) protein levels, methylation of H3 acetylated at Lys9 protein levels, as well as several deacetylases gene expression and/or protein levels, such as HDAC2, Sirt1, Sirt2, and Sirt6. CMS decreased H3 acetylation in senescence-resistant mice, similarly to SAMP8 control group, and increased in SAMP8 mice (Figure 1A). Considering H4, no statistically significant differences were determined, although a tendency to diminish in SAMR1 was observed (Figure 1B). On one hand, in accordance to H3 acetylation levels, there was a huge difference in histone deacetylase 2 (HDAC2) protein levels between SAMR1 and SAMP8 control groups, being higher in SAMP8 (Figure 1C). On the other hand, CMS modified HDAC2 protein levels, particularly decreased in SAMP8 and increased in SAMR1 compared to their littermates. Other deacetylases enzymes are of the sirtuin (Sirt) family. Not only gene expression (Figure 1D,E), but also protein levels of Sirt1, Sirt2 and Sirt6 were clearly decreased in SAMP8 mice compared to their control strain (SAMR1). In addition, CMS reduced *Sirt1* (Figure 1D) and *Sirt2* (Figure S1) gene expression and protein levels (Figure S1) in SAMR1 mice, but not in SAMP8. Moreover, histones can suffer different modifications such as phosphorylation or methylation, among others. In our hands, p-H2A-X protein levels did not differ between SAMR1 and SAMP8 control groups. Methylation of H3K9 was also higher in SAMP8 (Figure 1G). Of note, CMS increased the phosphorylated form of histones p-H2A-X and methylation of H3K9 in SAMP8 in reference to their control littermates, as well as compared to stressed SAMR1 (Figure 1F,G).

**Figure 2:**
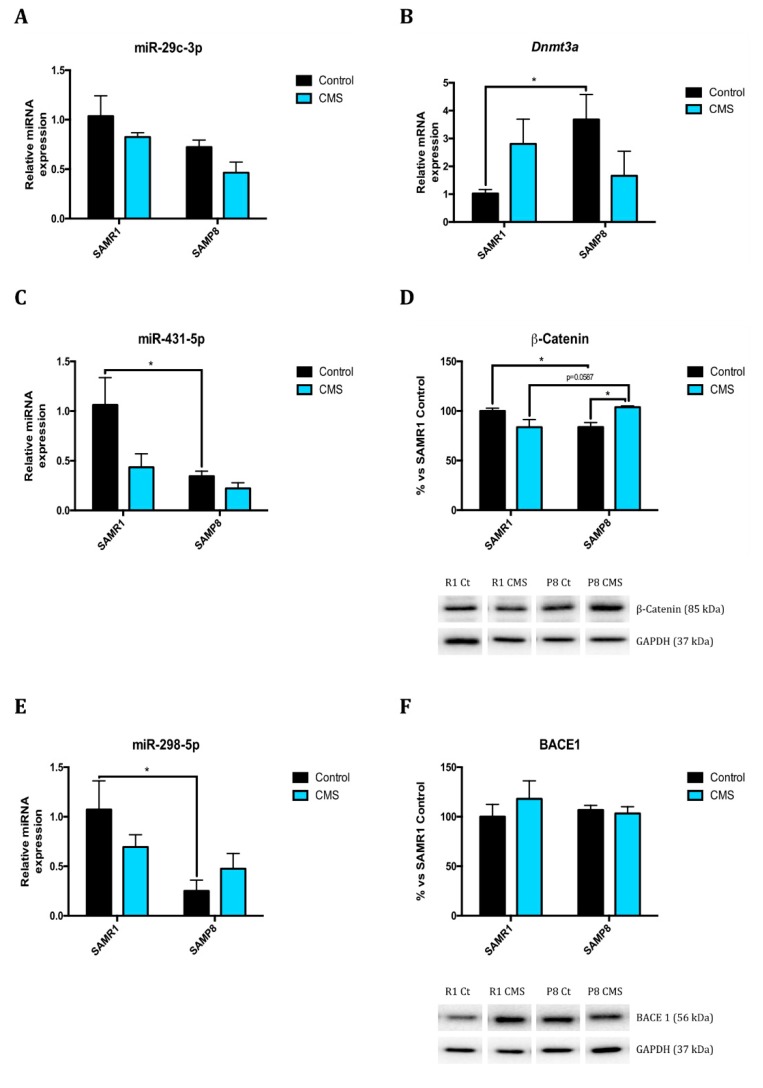

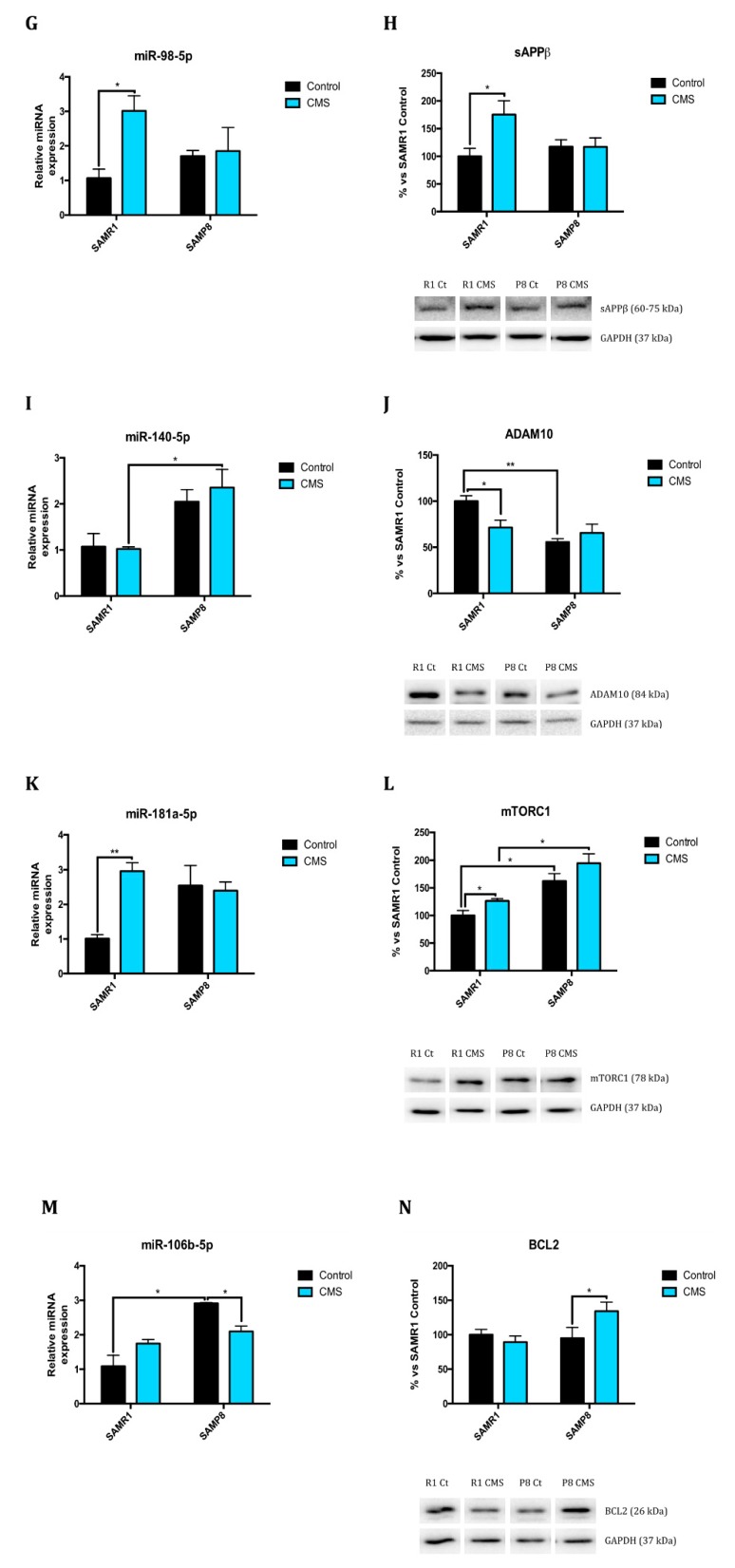
Validation of a representative subset of miRNA involved in brain aging, neurodegeneration and autophagy. Relative expression of miR-29c-3p, miR-431-5p, miR-298-5p, miR-98-5p, miR-140-5p, miR-181a-5p, and miR-106b-5p in the hippocampus of SAMR1 and SAMP8 mice (**A**,**C**,**E**,**G**,**I**,**K**,**M**). Relative gene expression of *Dnmt3a* (**B**) and representative Western Blot for β-Catenin protein levels and quantification (**D**), BACE1 protein levels and quantification (**F**), sAPPβ protein levels and quantification (**H**), ADAM10 protein levels and quantification (**J**), mTORC1 protein levels and quantification (**L**), and BCL2 protein levels and quantification (**N**). Gene expression levels were determined by real-time PCR. Values in bar graphs are adjusted to 100% for protein levels of SAMR1 control (R1 Ct). Values are mean ± Standard error of the mean (SEM); (*n* = 6 for each group in miRNAs validation; *n* = 4 for each group in WB and real-time PCR studies). * *p* <0.05; ** *p* < 0.01.

Considering DNA methylation, global 5-mC DNA was slightly lower in SAMP8 compared to SAMR1. CMS significantly reduced global methylation both in SAMP8 and SAMR1 (Figure 1H). DNA methylation occurs through DNA MethylTransferase (DNMT) enzymes, of which DNMT1, DNMT3A and DNMT3B are the best characterized. As described, DNMTs were significantly higher expressed in SAMP8 compared to SAMR1 control group in CMS animals (Figure 2B for *Dnmt3;* data not shown for *Dnmt1* and *Dnmt2*). 5-mC can be further oxidized to 5-hydroxymethyl-cytosine (5-hmC) due to ten-eleven translocase (TET) enzymes. SAMP8 mice had lower 5-hmC levels than SAMR1 mice (Figure 1I). Accordingly, TET2 protein levels were lower in SAMR1 than in SAMP8. CMS reduced TET2 protein levels in SAMR1 up to levels observed in SAMP8 control group (Figure 1J).

miRNA also play an important role in regulating gene expression. Thereby, we decided to perform a microarray with 84 miRNAs related with neurodegeneration and aging in order to evaluate the influence of our experimental conditions (strain and CMS) those miRNA expression. Considering these results (Tables S3 and S4), we proceeded to validate the relative expression of those modified miRNA in a significant manner regarding strain and CMS.

miR-29c is involved in neural proliferation regulation by Dnmt3. Although no statistical differences were seen, it seems that SAMP8 has higher relative expression than SAMR1, and CMS contributed to increasing it (Figure 2A,B). Those changes correlate with the tendencies seen in miR-29c-3p relative expression. miR-431-5p, miR-298-5p, miR-98-5p, and miR-140-5p expression were lower in SAMP8 than in SAMR1 (Figure 2C,E,G,I). Those miRNA are related to the inhibition of neurodegenerative pathways; its expression diminution correlated with changes in effector protein expression as β-catenin, β-site APP Cleaving Enzyme 1 (BACE1), soluble amyloid precursor protein-β (sAPPβ), and A Disintegrin and Metalloproteinase Domain-containing protein 10 (ADAM10) (Figure 2D,F,H,J). Furthermore, CMS seemed to reduce miR-431-5p expression in SAMR1 group, although not statistical differences were observed (Figure 2C,D). miR-181a-5p expression was higher in SAMP8 than SAMR1 (Figure 2K). Mammalian Target Of Rapamycin Complex 1 (mTORC1) is targeted by this miRNA, regulating cell growth, proliferation and survival. Likewise, mTORC1 protein levels were significantly higher in SAMP8 than in SAMR1 mice (Figure 2L). Stressful stimuli only increased miR-181a-5p relative expression in SAMR1 mice compared to their control littermates but not in SAMP8 (Figure 2K). Correlating with miRNA expression, the SAMR1-stressed group showed higher mTORC1 protein levels than SAMR1 control (Figure 2L). BCL2 protein levels are controlled by miR-106b-5p. SAMP8 mice showed higher relative expression in comparison with SAMR1 animals, and CMS reduced its expression only in SAMP8 strain (Figure 2M), which correlated with BCL2 protein levels (Figure 2N).

### 2.2. Chronic Mild Stress Attenuates Antioxidant Defence Mechanisms

**Figure 3:**
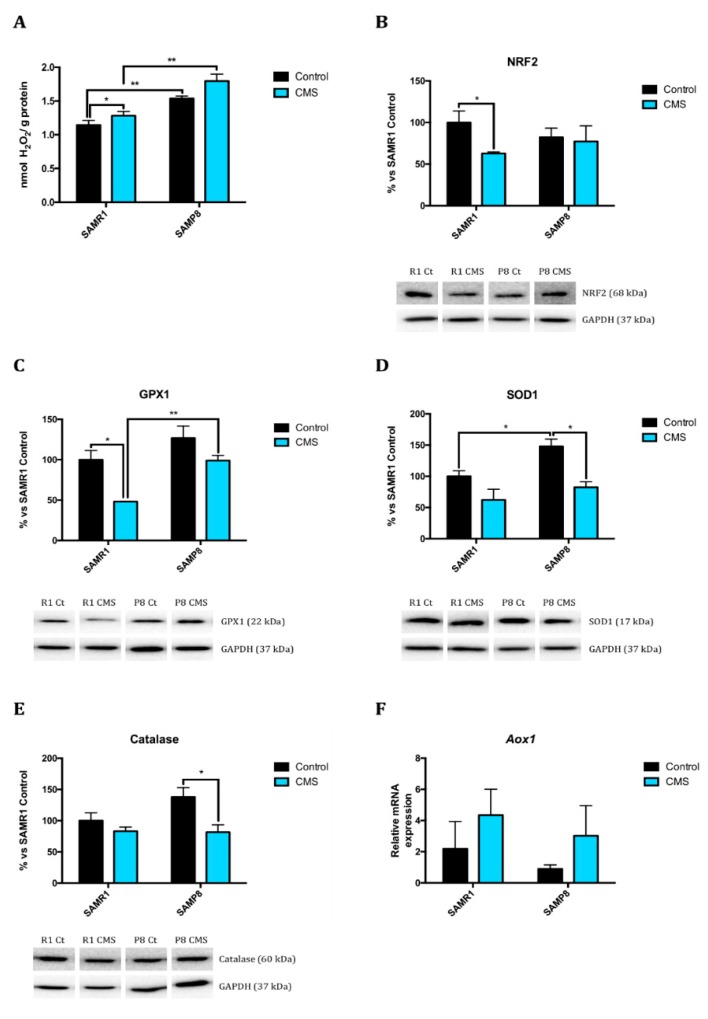
Changes in cellular oxidative stress after CMS. Representative OS measured as hydrogen peroxide concentration in homogenates of hippocampus tissue (**A**). Representative Western Blot for NRF2 protein levels and quantification (**B**), GPX1 protein levels and quantification (**C**), SOD1 protein levels and quantification (**D**), and Catalase protein levels and quantification (**E**). Relative gene expression of *Aox1* (**F**). Values in bar graphs are adjusted to 100% for protein levels of SAMR1 control (R1 Ct). Gene expression levels were determined by real-time PCR. Values are mean ± Standard error of the mean (SEM); (*n* = 4 for each group). * *p*< 0.05; ** *p* < 0.01.

As mentioned before, CMS modulated cell oxidative stress mechanisms favouring an oxidative state. SAMP8 animals showed higher H_2_O_2_ levels in the hippocampus than SAMR1 groups, as well as increased protein levels of the antioxidant enzymes superoxide dismutase 1 (SOD1), catalase (CAT) and glutathione peroxidase 1 (GPX1), suggesting impaired mechanisms to fight against OS (Figure 3A–E). CMS induced a diminution in Nuclear factor erythroid 2-related factor 2 (NRF2) protein levels only in SAMR1, but reduced antioxidant enzymes studied in both strains. In addition, CMS produced a slight but not significant tendency to increase aldehyde oxidase 1 (*Aox1*) gene expression, a pro-oxidant enzyme, in both strains (Figure 3F).

### 2.3. Inflammatory Activation is Induced after Application of Chronic Mild Stress

**Figure 4:**
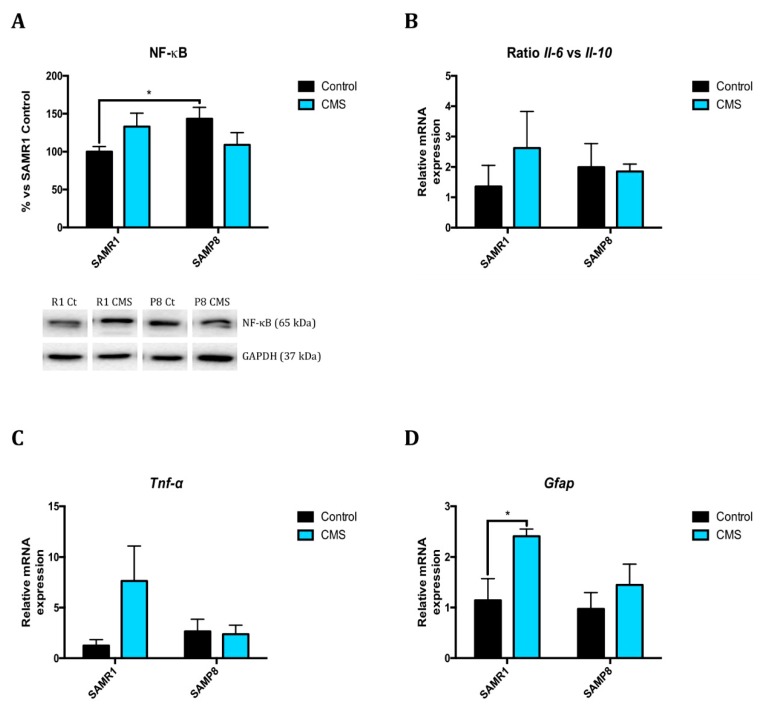
CMS induced changes in inflammatory markers. Representative Western Blot for NF-κB protein levels and quantification (**A**). Relative gene expression of *Il-6/Il-10* ratio, *Tnf-α* and *Gfap* (**B**–**D**). Values in bar graphs are adjusted to 100% for protein levels of SAMR1 control (R1 Ct). Gene expression levels were determined by real-time PCR. Values are mean ± Standard error of the mean (SEM); (*n* = 4 for each group). * *p* < 0.05.

SAMP8 mice show higher protein levels of NF-κB compared to the SAMR1 control group. CMS seems to promote higher cytokines expression in SAMR1 than in SAMP8, as shown in the ratio *Interleukin(Il)-6/Il-10* and *tumor necrosis factor (Tnf) α* levels (Figure 4A–C), although no statistical differences were found. In accordance, *Glial fibrillar acidic protein (Gfap)* gene expression increased in the SAMR1 CMS group, and there is a slight increase in the SAMP8 CMS group in comparison to respective controls (Figure 4D).

### 2.4. Chronic Mild Stress Modulates APP Processing as Well as Alters Microtubule Stabilization Through Hyperphosphorylation of Tau Protein

**Figure 5:**
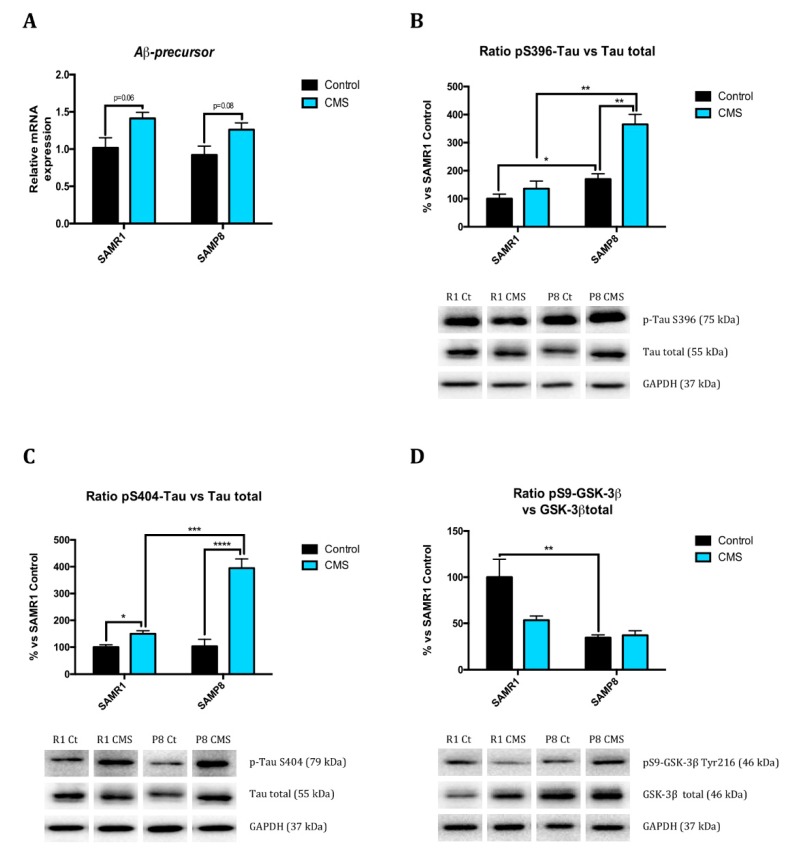
AD hallmarks changes after CMS. Relative gene expression of *Aβ-precursor* (**A**). Representative Western Blot for the ratio of pS396-Tau protein levels and quantification (**B**), the ratio of pS404-Tau protein levels and quantification (**C**), and the ratio of pS9-GSK-3β protein levels and quantification (**D**). Gene expression levels were determined by real-time PCR. Values in bar graphs are adjusted to 100% for protein levels of SAMR1 control (R1 Ct). Values are mean ± Standard error of the mean (SEM); (*n* = 4 for each group). * *p* < 0.05; ** *p* < 0.01; *** *p* < 0.001; **** *p* < 0.0001.

Observing the results, SAMP8 showed lower ADAM10 protein levels than SAMR1 animals. CMS promoted the β-amyloid amyloidogenic pathway in SAMR1 mice, increased sAPPβ protein levels and BACE1 protein levels, and there was a tendency to increase Aβ-precursor gene expression as well as reduce ADAM10 protein levels (Figure 2F,H,J and Figure 5A).

SAMP8 had higher Ser396 p-Tau levels (Figure 5B,C) than SAMR1. Similarly, GSK-3β phosphorylation at Ser9 protein level was lower in SAMP8 in reference to SAMR1 (Figure 5D). CMS increased tau hyperphosphorylation at Ser396 and Ser404 compared to control groups; surprisingly, there is a trend towards CMS reducing GSK-3β protein levels (Figure 5D).

### 2.5. Chronic Mild Stress Promotes Autophagy Activation

**Figure 6:**
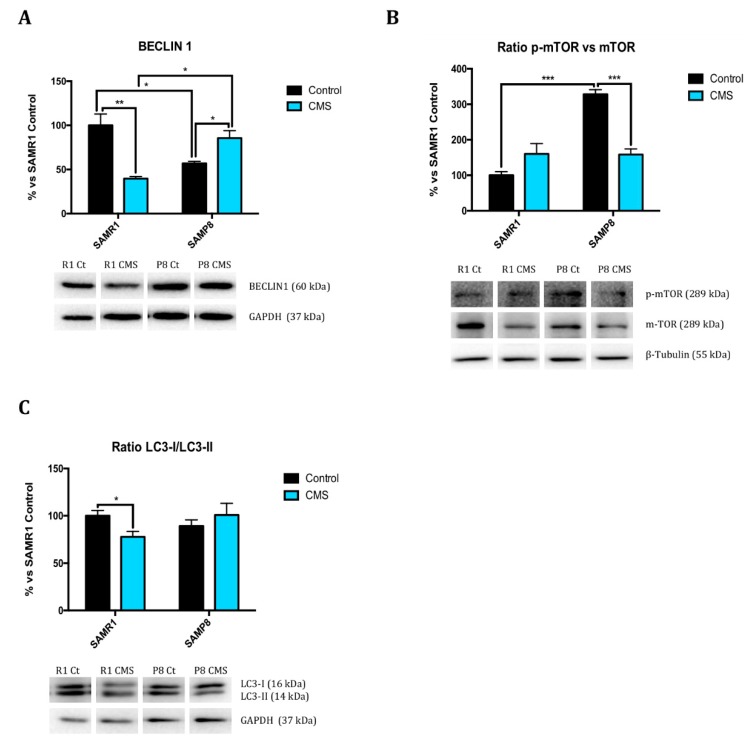
CMS induced autophagy flow modifications in SAMR1 and SAMP8. Representative Western Blot for Beclin 1 protein levels and quantification (**A**), the ratio of p-mTOR protein levels and quantification (**B**) and the ratio of LC3 protein levels and quantification (**C**). Values in bar graphs are adjusted to 100% for protein levels of SAMR1 control (R1 Ct). Values are mean ± Standard error of the mean (SEM); (*n* = 4 for each group). * *p* < 0.05; ** *p* < 0.01; *** *p* < 0.001.

Because autophagy has a key role in neurodegeneration, we evaluated Beclin 1, microtubule-associated protein 1A/1B-Light Chain 3 (LC3B), B-Cell Lymphoma 2 (BCL2), and mammalian Target Of Rapamycin (mTOR). Beclin 1 and LC3B protein levels were lower in SAMP8 than in SAMR1, whereas mTOR activation, measured by p-mTOR/mTOR ratio, was higher in SAMP8 mice. CMS decreased the pro-autophagic protein levels studied (Beclin 1 and LC3B) in SAMR1 mice, as well as there was a slight increase in mTOR activation. By contrast, in SAMP8 animals, stressful stimuli increased the majority of pro-autophagy and decreased anti-autophagy markers evaluated (Figure 6A–C; Figure 2N).

### 2.6. Anxiety-Like Behaviour, Memory Decline and Poorer Cognitive Abilities After Chronic Mild Stress

**Figure 7:**
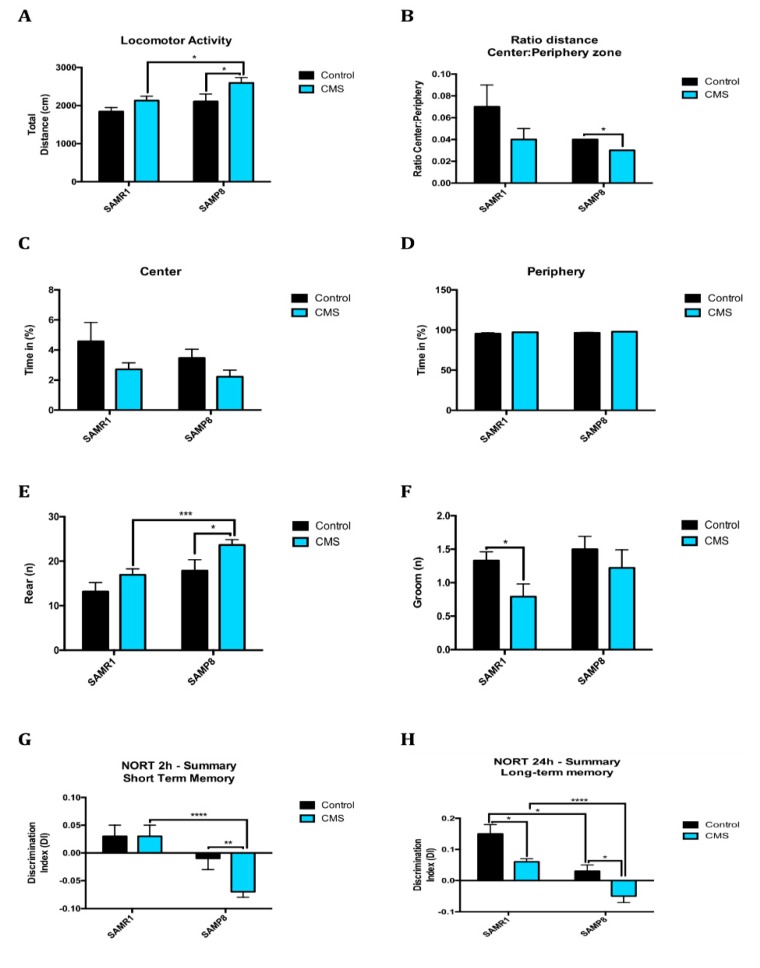

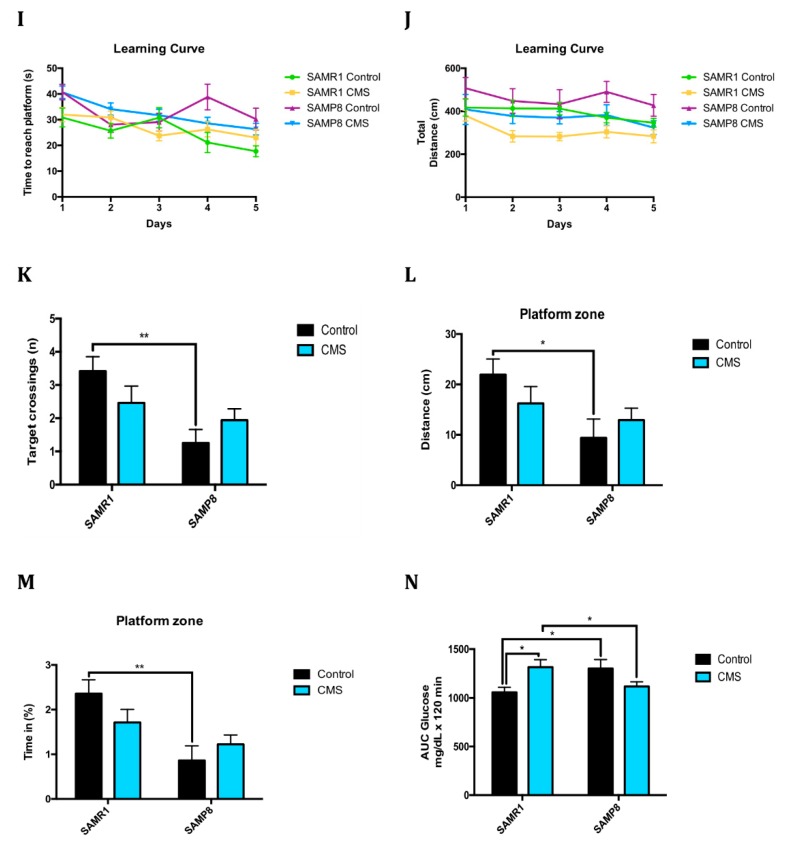
Behavioural and cognitive paremeters after CMS in SAMR1 and SAMP8 mice. Results of OFT for all mice groups. Locomotor Activity (**A**), Ratio of the distance travelled in the Center/Periphery zone (**B**), Time in the Center (**C**) and in the Periphery (**D**), number of Rearing (**E**), and number of Grooming (**F**). Results of NORT for all mice groups. Summary of DI from 2 and 24 h after familiarization phase (**G**,**H**). Results of MWM for all mice groups. Learning curves of MWM during the spatial acquisition phase (**I**,**J**), number of Entries (**K**), Distance travelled (**L**), and Time (**M**) in platform zone during the test. Plasma levels of glucose 2 g/kg intraperitoneal (i.p.) administration (**N**). Values are mean ± Standard error of the mean (SEM) (*n* = 14 for each group). * *p* < 0.05; ** *p* < 0.01; *** *p* < 0.001; **** *p* < 0.0001.

Stress produced behavioural changes in animals. In particular, the CMS treatment applied to animals contributed to increase locomotor activity in both mice strains, indicating that anxiety sensation and escape desire increased (Figure 7A). Regarding the other parameters evaluated in the OFT, SAMR1 travelled more distance and stood longer in the centre compared to SAMP8, although there were no statistically significant differences. In addition, CMS reduced those parameters in both strains compared to control animals (Figure 7B–D). Among these parameters indicating anxious behaviour were grooming and rearing. CMS-treated groups showed a higher number of rears and lower number of grooms than their controls (Figure 7E,F).

Referring to memory, we evaluated the recognition and spatial memory as well as learning abilities, through NORT and MWM. On one hand, there was a clear difference between strains because SAMP8 groups showed lower recognition memory than SAMR1 mice. On the other hand, MWM learning curves demonstrated that all mice learned where the platform was during the training days, as the time to reach the platform and the distance travelled was lower than the first day (Figure 7I,J). CMS treatment reduced recognition memory in both the short and long term (2 h and 24 h) (Figure 7G,H). Considering the results obtained in MWM, SAMR1 animals crossed the platform zone and its quadrant more times than the SAMP8 mice, as well as they stood and swum more distance in the platform zone (Figure 7K–M), indicating a significantly better performance in this maze from SAMR1 than SAMP8. CMS decreased the value of those parameters in SAMR1 bringing them closer to SAMP8 values, although they did not reach significance. Overall, results obtained in MWM and in accordance to OFT results indicated that CMS increased the total distance travelled during the training in comparison with control groups.

Lastly, glucose metabolism was evaluated through a glucose tolerance test. SAMP8 mice showed higher glucose area under the curve than SAMR1 mice, although this relation was upside down between animals that received CMS (Figure 7N), suggesting that stressed SAMR1 mice develop a sugar metabolism similar to SAMP8 animals.

## 3. Discussion

Understanding the mechanisms that define aging and differentiate what determines whether it is pathologic or healthy is one of the challenges that science has faced in recent years. Life expectancy has increased and so has the number of older people. In addition, the life rhythm has changed, becoming increasingly stressful. It has been shown that the environment is extremely important in the development of several diseases, compromising healthy aging; specifically, stressful lifestyle has been identified as an important risk factor for cognitive decline. Therefore, it is crucial to study the effects of stress on cognition and its relationship with aging in order to unveil what challenges we might have to cope with as a society in the not-so-far future. We hypothesize that chronic stress would modulate a large constellation of cellular mechanisms implied in aging and age-related neurodegenerative pathologies.

During aging, there are several epigenetic mechanisms altered, which include DNA methylation, histone modifications, nucleosome remodelling, and miRNA-mediated gene regulation [2,23]. In the present study, we evaluated three main epigenetic marks, which modulate chromatin structure and act as platforms for recruitment, assembly or retention of chromatin-associated factors: histone posttranslational modifications, DNA methylation and miRNA-mediated gene regulation. One of the posttranslational modifications of histones is acetylation. This process removes the histone positive charge; thereby, the condensed chromatin (heterochromatin) is transformed into a more relaxed structure (euchromatin) that is associated with greater levels of gene transcription [24,25]. However, chromatin condensation depends on different processes including methylation and histone deacetylation. This last process is due to histone deacetylases (HDAC) enzymes. Deregulation of histone acetylation has been related to an increase of the risk of age-dependent memory impairment in mice [4,26,27]. Specifically, histone H4 lysine 12 acetylation alterations causes impaired memory consolidation as well as its restoration reinstates the expression of learning-induced genes and in consequence, cognitive abilities [26]. In accordance to Cosín-Tomás [28], we found that CMS increased HDAC2 protein levels only in SAMR1 females, similarly to SAMP8 control mice, which lead us to hypothesize that acetylated histone protein levels were diminished. This was confirmed evaluating acetylated H3 and H4 protein levels. However, while stress produced changes in H3 acetylation, these changes were not observed in H4. Nevertheless, the decrease in H3 lysine 9 (H3K9) acetylation in SAMR1 mice under CMS, similarly to not stressed SAMP8, was correlated with changes on cognition. Other HDACs include sirtuins family; however, it is worth noting that several members of this family do not have deacetylase activity [29]. Sirt have been linked to aging as they modulate genomic stability, stress resistance and energy metabolism. Activation of Sirt1 enables the deacetylation of a variety of proteins, resulting in a robust, protective cellular response, as it regulates processes such as cell death, metabolism or neurodegeneration [30]; while Sirt2 has been reported to regulate oxidative stress, genome integrity and myelination and its dysfunction is found in most age-related neurodegenerative disorders such as AD, Parkinson’s disease and Amyotrophic Lateral Sclerosis, as well as in physiological aging [31]. Accumulated evidence indicates that *Sirt6* gene expression is lower in the hippocampus and cerebral cortex of aged mice [32,33] and that it is concerned with H3K9 acetylation [32]. In reference to this family of deacetylases, our results demonstrate decreased *Sirt1, Sirt2* and *Sirt6* gene expression in SAMP8 mice compared to SAMR1 and SAMR1 under CMS. Compelling evidence has proposed that methylation of H3K9 promotes DNA methylation maintenance in mammals and is a hallmark of heterochromatin formation and subsequent gene silencing [2,34,35]. As with histone phosphorylation, CMS treatment increased H3K9 methylation in SAMP8 animals but not in SAMR1, suggesting that even they showed more H3 acetylation, which also resulted in more methylation. Therefore, we demonstrate that CMS favoured chromatin condensation and in consequence, promoted gene silencing.

In reference to other modifications, histone phosphorylation belongs to the cellular response to DNA damage, as phosphorylated histone H2A.X demarcates large chromatin domains around the site of DNA breakage [2]. Additionally, multiple studies have also shown that histone phosphorylation plays crucial roles in other nuclear processes, such as DNA replication because of apoptosis or DNA damage [36]. Here CMS raised phosphorylated H2A.X protein level in senescence-accelerated but not in senescence-resistant mice, suggesting that stressful stimuli activate repair/survival mechanisms rather than apoptotic response in SAMP8 mice but not in SAMR1. As two major mechanisms for epigenetic regulation, DNA methylation and histone modifications must act coordinately [34]. It is well known that DNA methylation at the fifth position of cytosine (5-mC) plays an important role in neuronal gene expression and neural development. Several studies support the idea that dysregulated DNA methylation/demethylation is linked to many neuronal disorders, including AD onset and progression. However, the relationship between AD and altered 5-mC levels is not known [37]. 5-mC can be further oxidized to 5-hmC, among others, by the TET family of dioxygenases. 5-mC and 5-hmC exert opposite effects on gene expression; the former is in general associated with gene silencing, whereas the latter is mainly involved in up-regulation of gene expression [38]. In this study, the stressful environment produced lower 5-mC in both mouse strains and differences between strains were observed in 5-hmC marker. Accordingly, TET2 protein levels differed between SAMR1 and SAMP8 animals. Despite our previous results, conversely 5-mC and 5-hmC results appear to contradict the transcriptional access to DNA, but as is known, the term global DNA methylation describes this process across the entire genome and does not represent a precise landscape for specific transcriptional activity; however, global methylation determination is useful as it provides an over-arching picture of methylation status; it is misleading which genes show altered DNA methylation and which do not [39].

Furthermore, we evaluated expression changes of miRNAs related to oxidative stress and cellular pathways implicated in AD neuronal death, autophagy and neurodegeneration. Of the 22 miRNA evaluated, those that presented statistically significant changes under experimental conditions were further validated and target genes studied (Figure S2). Firstly, we studied miR-29c relative expression, which is involved in neural proliferation regulation through Dnmt3a. *Dnmt3a* expression was higher in SAMP8 compared to SAMR1 mice correlating with miR-29c-3p gene expression. In our hands, CMS slightly contributed to change *Dnmt3a* expression in mice, showing a trend towards an increase in SAMR1. Because recent studies demonstrate that DNMT inhibitors provided neuroprotection in cellular cultures [40,41], the increase in *Dnmt3a* induced by CMS could mean a deleterious effect on SAMR1 health.

Considering AD neuropathology, we studied miR-431-5p, miR-298-5p, miR-98-5p, and miR-140-5p. It has been described that miR-431-5p cooperates with DKK1 to inhibit Wnt/β-catenin pathway. SAMP8 showed lower relative expression of this miRNA in comparison to SAMR1 mice; changes in β-catenin protein levels were in line with miR-431-5p. Furthermore, there is a tendency to reduce miR-431-5p gene expression and β-catenin protein levels in SAMR1 under CMS. β-catenin is regulated by GSK-3β activity, depending on its phosphorylation. As reported, GSK3-β inactive form (Ser9 phosphorylated) protein levels were lower in SAMP8 in reference to SAMR1 [30], and also CMS decreased them, especially in SAMP8. It is known that GSK3-β activity is associated with AD neuropathology as it exacerbates cognitive impairment [42]. In fact, higher activity of this kinase has been found in AD patients and its inhibition restores spatial memory deficits, reduces tau hyperphosphorylation, and decreases reactive gliosis and neuronal death in rodents [42]. Furthermore, it has been described that GSK3-β inhibition reduces BACE1-mediated cleavage of APP through NF-κB signalling-mediated mechanism, so that it reduces β-amyloid pathology [43]. Accordingly, we found that CMS increased Ser396 and Ser404 Tau hyperphosphorylation, most pronounced in SAMP8, and promoted amyloid precursor protein (APP) gene expression in both mice strains. In fact, APP processing is regulated by miR-298-5p, which in turn regulates BACE1. miR-298-5p expression differed between SAMR1 and SAMP8 animals, in contrast with BACE1 protein levels. It is reported that BACE1, sAPPβ and β-CTF protein levels were increased when miR-98-5p up-regulated. CMS increased miR-98-5p relative expression and sAPPβ in SAMR1 compared to the control group, but not in SAMP8. However, as mentioned, BACE1 was not modulated under CMS. Taking into account that BACE1 can be up- or down-regulated by different miRNAs, discrepancies can be explained by compensatory responses among different signals. ADAM10 protein levels are controlled by miR-140-5p. Again, huge differences in miR-140-5p gene expression and ADAM10 protein levels were observed between strains. There were differences between control mice under CMS in SAMR1 but not in SAMP8.

miR-181a-5p is involved in the regulation of cell growth, proliferation and survival through mTOR complex 1 (mTORC1) and downstream pathway, in which protein levels were increased in SAMP8 in comparison to SAMR1 mice. In addition, stressful stimuli increased miR-181a-5p relative expression in SAMR1 mice compared to their control littermates and no changes were observed between SAMP8 animals. By contrast, CMS groups in both strains showed higher mTORC1 protein levels than their respective control littermates. Lastly, BCL2 protein levels were controlled by miR-106b-5p. SAMP8 mice had higher miR-106b-5p compared to SAMR1, but no changes were found in BCL2 protein levels. CMS reduced miR-106b-5p gene expression, increasing BCL2 protein levels in SAMP8, without affecting SAMR1 strain.

Autophagy declines during aging, so that it may contribute to the deleterious accumulation of aberrant proteins observed in aged cells. Interestingly, failure of this process has been reported to worsen aging-associated diseases, such as neurodegeneration or cancer [18]. Pro-autophagy proteins such as Beclin1 and LC3B became decreased after CMS treatment, and in concordance, the p-mTOR ratio was increased. Moreover, we demonstrate huge differences between SAMR1 and SAMP8 control groups, indicating a reduced autophagic flux in senescent mice. CMS-induced impairment in autophagy was more robust and consistent in SAMR1 than in SAMP8, supporting the hypothesis that CMS accelerates the senescence process.

Following the same line about overall processes linked to senescence, it has been widely described that OS possesses a pre-eminent role in pathological senescence and the pathogenesis of AD. In general, cells possess antioxidant mechanisms to cope with OS, such as GPX, Catalase and SOD1, among others, which in turn are regulated by the Nrf2 transcriptional pathway. Herein we found that mice under CMS had lower NRF2 protein levels than control groups and this in turn could explain the lowest protein levels of antioxidant defence as GPX1, SOD1 and Catalase. Consistent with this, CMS promoted an increase in the pro-oxidant enzyme *Aox1* gene expression and higher accumulation of ROS. Noteworthy, as described, differences between strains were found in most of the markers evaluated [4].

As far as aging and AD are concerned, dysregulation of inflammatory mediators and astrogliosis are major culprits in the development of chronic inflammation and the immunosenescence process, as well as are related to cognitive decline and progression of neurodegenerative diseases [15]. For instance, our results demonstrate a significant increase in protein levels for NF-κB, transcription factor regulating pro-inflammatory signals, in SAMP8 mice in comparison with SAMR1. One of these signaling pathways includes the insulin and insulin-like growth factor (IGF) pathways; 5’-AMP-activated protein kinase (AMPK)-mechanistic target of rapamycin (mTOR) pathway; and Forkhead box O (FOXO) families, sirtuin (SIRT), and p53-related pathways [15]. Interestingly, CMS increased proinflammatory pathways in SAMR1 but not in SAMP8. Moreover, we found an increase in *Gfap* gene expression in SAMR1 CMS compared to SAMR1 control group. In accordance, recently it has been described that decreasing astrogliosis enhances AD pathology in mice [44].

The detrimental effect of stress on psychological well-being and cognitive functioning, emphasizing the relationship between stress and memory, is widely accepted. It is noteworthy that during stressful conditions underlie some of the characteristics described in cognitive decline, either in aging or in neurodegenerative diseases, such as AD [19,45]. It has been stated that epigenetic machinery is essential for cognitive function [2,26,46]. Likewise, DNA methylation influences hippocampal memory formation, and growing evidence suggests that the modulation of epigenetic processes by stress, EE and/or hormones is key in regulating memory function.

In line with the molecular results presented, behavioural changes induced by CMS were explored in SAMP8 and SAMR1 mice. Results obtained point out that not only a stressful environment triggers anxiety-like behaviour, but also it mitigates cognitive performance. Locomotor activity, the distance travelled in the central zone and other parameters, indicates well-being/discomfort were altered in mice under CMS. Regarding recognition memory, clear differences were found between the different strains, although we can also assert a detrimental effect caused by CMS, especially on long-term memory. In agreement with these results, learning abilities and spatial memory in SAMR1 and SAMP8 at 6 months of age were significantly different and were negatively affected by the presence of chronic stressors. In stressful situations, glucocorticoids are released into the bloodstream causing, among others, an increase in glucose level in order to have enough energy available to cope with stress. Aberrant glucose metabolism potentiates the aging phenotype and contributes to early stage central nervous system pathology [47]. According to previous studies, we found differences in blood glucose levels due to stress in SAMR1 mice, exhibiting similar glucose tolerance to SAMP8 control mice. This observation means that the CMS paradigm applied was enough to induce a stressful condition in mice, facilitating an acceleration of the aging process in SAMR1.

## 4. Materials and Methods

### 4.1. Animals and Chronic Mild Stress Procedure

Female SAMP8 mice (*n* = 56) were used to perform behavioural, cognitive and molecular analyses. We divided these animals into four groups: SAMR1 (SAMR1 control, *n* = 14), SAMR1 treated with CMS (SAMR1 CMS, *n* = 14), SAMP8 (SAMP8 control, *n* = 14) and SAMP8 treated with CMS (SAMP8 CMS, *n* = 14). Animals had free access to food and water and were kept under standard temperature conditions (22 ± 2 °C) and 12 h:12 h light–dark cycles (300 lux/0 lux). Starting at 4 months of age, CMS groups received the Chronic Mild Stress (CMS) treatment. The CMS procedure that was used in the present study had previously been validated in SAMR1 and SAMP8 mice, with some modifications [22]. Mice were exposed to various randomly scheduled, low-intensity environmental stressors every day for 4 weeks. Different stressful stimuli were applied every day and the sequence of the stressors was altered every week to guarantee the degree of unpredictability. Among others, CMS stimulus were 24 h of water deprivation, 24 h of food deprivation, 2 h of physical restraint, 24 h of sawdust removal, 24 h of wet bedding, overnight illumination, and 1 min of tail nipping at 1 cm from the tip of the tail. Before the performance of the cognitive tests, the glucose tolerance test was conducted.

Studies and procedures involving mouse brain dissection and subcellular fractionation were performed following the institutional guidelines for the care and use of laboratory animals established by the Ethical Committee for Animal Experimentation at the University of Barcelona.

### 4.2. Glucose Tolerance Test

Intraperitoneal (i.p.) glucose tolerance test was performed following 4 weeks of CMS treatment, as described previously. In brief, mice were fasted overnight for 12 h. The test was performed in a quiet room and 2 g/kg i.p. glucose injection was administered (diluted in H_2_O) and blood glucose levels were measured at 0, 5, 10, 15, 30, 60, and 120 min after the injection with the Accu-Chek^®^ Aviva blood glucose meter (Accu-Chek^®^ Aviva, Roche, Barcelona, Spain).

### 4.3. Behavioural and Cognitive Test

#### 4.3.1. Open Field Test (OFT)

The OFT was performed as previously described in Griñán-Ferré et al. [48]. It evaluates anxiety-like behaviour. Mice were placed at the centre of a white polywood box (50 × 50 × 25 cm) and allowed to explore it for 5 min. Behaviour was scored with SMART^®^ ver.3.0 software, and each trial was recorded for later analysis. The parameters scored included centre staying duration, rearing, grooming, and the distance travelled.

#### 4.3.2. Novel Object Recognition Test (NORT)

The Novel Object Recognition Test (NORT) protocol employed was as described in Puigoriol-Illamola et al. [49]. In brief, mice were placed in a 90°, two-arms, 25 cm-long, 20 cm-high, 5 cm-wide black maze. Before performing the test, the mice were individually habituated to the apparatus for 10 min for 3 days. On day 4, the animals were submitted to a 10 min acquisition trial (first trial), during which they were placed in the maze in the presence of two identical, novel objects at the end of each arm. After a delay (2 h and 24 h), the animal was exposed to two objects, one old object and one novel object. The Time that mice explored the Novel object (TN) and Time that mice explored the Old object (TO) were measured. A Discrimination Index (DI) was defined as (TN − TO)/(TN + TO). To avoid object preference biases, objects were counterbalanced. The maze, surface, and objects were cleaned with 70% ethanol between the animals’ trials to eliminate olfactory cues.

#### 4.3.3. Morris Water Maze (MWM)

This test evaluates both learning and spatial memory [50]. An open circular pool (100 cm in diameter, 50 cm in height) filled with water was used. Water was painted white with latex in order to make it opaque and its temperature was 22 ±  1 °C. Two main perpendicular axes were established (North-South and East-West), thus configuring four equal quadrants (NE, NW, SE, and SW). Four visual clues (N, S, E, W) were placed on the walls of the tank so that the animal could orientate and fulfil the objective. The test consisted of training a mouse to find a submerged platform (Learning phase) and assess whether the animal has learned and remembered where the platform was the day that it was removed (Test). The training lasted 5 consecutive days, and every day, five trials were performed, which have different starting points (NE, E, SE, S, and SW), with the aim that the animal recognizes the visual clues and learns how to locate the platform, avoiding learning the same path. At each trial, the mouse was placed gently into the water, facing the wall of the pool, allowed to swim for 60 s, and there was not a resting time between trials. If the animal was not able to locate the platform, the investigator guided it to the platform and was allowed to rest and orientate for 30 s. The platform was placed approximately in the middle of one of the quadrants, 1.5 cm below the water level. Above the pool there was a camera that recorded the animals’ swimming paths, and the data were analysed with the statistical program SMART^®^ ver.3.0. During the learning phase, a learning curve was drawn, in which is represented the latency to find the platform every training day. On the day test, more parameters were measured, such as the target crossings and the swum distance in the platform zone.

### 4.4. Immunodetection Experiments

#### 4.4.1. Brain Processing

Three days after the behavioural and cognitive tests, animals were euthanized for protein extraction, RNA and DNA isolation. brains were immediately removed, and the hippocampus was isolated, frozen on powdered dry ice, and maintained at −80 °C until procedures.

#### 4.4.2. Western Blotting

Tissue samples were homogenized in lysis buffer (Tris HCl pH 7.4 50 mM, NaCl 150 mM, EDTA 5 mM and 1X-Triton X-100) containing phosphatase and protease inhibitors (Cocktail II, Sigma-Aldrich) to obtain total protein homogenates. Aliquots of 15 μg of hippocampal protein extraction per sample were used. Protein samples were separated by Sodium dodecyl sulphate-polyacrylamide gel electrophoresis (SDS-PAGE) (8–14%) and transferred onto Polyvinylidene difluoride (PVDF) membranes (Millipore). Afterwards, membranes were blocked in 5% non-fat milk in Tris-buffered saline (TBS) solution containing 0.1% Tween 20 TBS (TBS-T) for 1 h at room temperature, followed by overnight incubation at a 4 °C with the primary antibodies listed in (Table S1). Then, the membranes were washed and incubated with secondary antibodies listed in (Table S1) for 1 h at room temperature. Immunoreactive proteins were viewed with the chemiluminescence-based ChemiLucent^TM^ detection kit, following the manufacturer’s protocol (ECL Kit, Millipore, Massachussets, USA), and digital images were acquired using ChemiDoc XRS + System (BioRad, California, USA). Semi-quantitative analyses were done using ImageLab software (BioRad, California, USA), and results were expressed in Arbitrary Units (AU), considering control protein levels as 100%. Protein loading was routinely monitored by immunodetection of Glyceraldehyde-3-phosphate dehydrogenase (GAPDH) or β-tubulin.

### 4.5. RNA Extraction and Gene Expression Determination by q-PCR

Total RNA isolation was carried out using TRIsure^TM^ reagent according to the manufacturer’s instructions (Bioline Reagent, UK). The yield, purity, and quality of RNA were determined spectrophotometrically with a NanoDrop^™^ ND-1000 (Thermo Scientific) apparatus and an Agilent 2100B Bioanalyzer (Agilent Technologies). RNAs with 260/280 ratios and RIN higher than 1.9 and 7.5, respectively, were selected. Reverse Transcription-Polymerase Chain Reaction (RT-PCR) was performed as follows: 2 μg of messenger RNA (mRNA) was reverse-transcribed using the High Capacity cDNA Reverse Transcription Kit (Applied Biosystems). Real-time quantitative PCR (qPCR) was used to quantify mRNA expression of oxidative stress and inflammatory genes listed in (Table S2). SYBR^®^ Green real-time PCR was performed in a Step One Plus Detection System (Applied-Biosystems) employing SYBR^®^ Green PCR Master Mix (Applied-Biosystems). Each reaction mixture contained 6.75 μL of complementary DNA (cDNA) (in which the concentration was 2 μg), 0.75 μL of each primer (which concentration was 100 nM), and 6.75 μL of SYBR^®^ Green PCR Master Mix (2×).

Data were analysed utilizing the comparative Cycle threshold (Ct) method (ΔΔCt), where the housekeeping gene level was used to normalize differences in sample loading and preparation [28]. Normalization of expression levels was performed with β-actin for SYBR^®^ Green-based real-time PCR results. Each sample was analysed in duplicate, and the results represent the n-fold difference of the transcript levels among different groups.

### 4.6. Global DNA Methylation and Hydroxymethylation Determination

Isolation of genomic DNA was conducted using the FitAmp^TM^ Blood and Cultured Cell DNA Extraction Kit (EpiGentek, Farmingdale, NY, USA) according to the manufacturer’s instructions. Following this, Methylflash Methylated DNA Quantification Kit (Epigentek, Farmingdale, NY, USA) and MethylFlash HydroxyMethylated DNA Quantification Kit were used in order to detect methylated and hydroxymethylated DNA. Briefly, these kits are based on specific antibody detection of 5-mC and 5-hmC residues, which trigger an ELISA-like reaction that allows colorimetric quantification at 450 nm.

### 4.7. miRNA Expression Array and Validation by Single Real-Time PCR

For microRNA (miRNA) expression array, total RNA and miRNA were extracted employing the miRNeasy Mini Kit (Qiagen) according to the manufacturer’s instructions. The yield, purity and quality of the samples were determined by the A260/280 ratio in a NanoDrop^®^ ND-1000 apparatus (Thermo Scientific). RNA samples from 24 females (*n* = 6 per group) were converted into cDNA through a Reverse Transcription (RT) reaction using the miRCURY^TM^ LNA^TM^ miRNA RT Kit (Exiqon) according to the manufacturer’s instructions. The expression of 22 mature miRNAs was then analysed using Mouse Pick&Mix miRNA PCR panel (Exiqon) (Tables S3 and S4). miRNA expression was measured in a StepOnePlus Real-Time PCR system (Applied Biosystems).

SYBR Green-based real-time PCR was performed on the detection system StepOnePlus (Applied Biosystems) for miRNA expression. In compliance with the miRCURY LNA^TM^ SYBR Green PCR Kit Protocol (Qiagen), each reaction mixture contained 4,5 μL of SYBR Green PCR Master Mix (2×), 0,5 μL of ROX, 3 μL of product from RT reaction diluted 1:60, 1 μL of nuclease-free water and 1 μL of miRNA probes (Exiqon) (Table S2). Data were analysed using the comparative cycle threshold (Ct) method in which the SNORD68 small non-coding RNA transcript level was employed to normalize differences, since it presented similar expression levels between groups. Each sample was analysed in duplicate and results represent the n-fold difference of the transcript levels among different groups.

### 4.8. Oxidative Stress Determination

Hydrogen peroxide was measured in hippocampus protein homogenates as an indicator of oxidative stress, and it was quantified using the Hydrogen Peroxide Assay Kit (Sigma-Aldrich, Saint Louis, MI, USA) according to the manufacturer’s instructions.

### 4.9. Data Analysis

Data analysis was conducted using GraphPad Prism ver. 7 statistical software. Data are expressed as the mean ± standard error of the mean (SEM) of at least six samples per group. Diet and treatment effects were assessed by the Two-Way ANOVA analysis of variance, followed by Tukey post-hoc analysis or two-tail Student’s *t*-test when it was necessary. Statistical significance was considered when *p*-values were <0.05. The statistical outliers were determined with Grubbs’ test and subsequently removed from the analysis when necessary.

## 5. Conclusions

**Figure 8:**
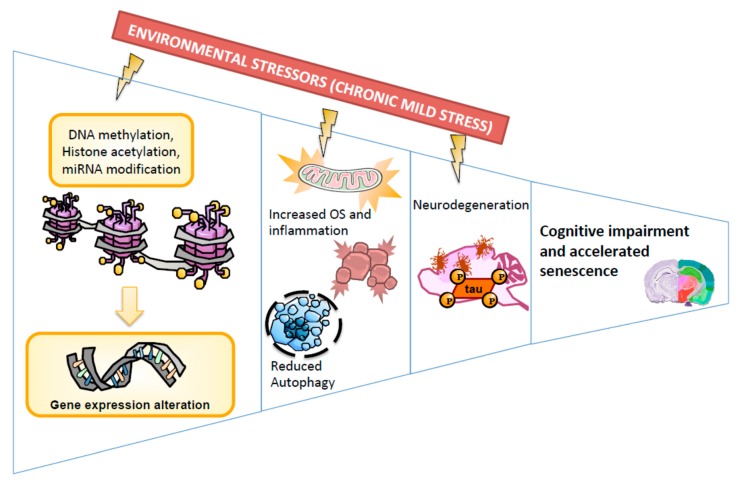
Representative scheme of molecular pathways altered after Chronic Mild Stress.

Overall, CMS treatment produces detrimental effects in SAM female mice, such as inflammatory signalling activation, loss of antioxidant defence mechanisms, changes in behaviour, and reduced cognitive abilities (Figure 8). Interestingly, CMS promoted significant epigenetic and biochemical changes in SAMR1 animals, a normal mice strain, driving them to an aging-specific phenotype represented by SAMP8 mice, a senescence mice mode, in which the negative effects of CMS were probably limited because its senescence level was already elevated.

## Supplementary Materials

Supplementary materials can be found at https://www.mdpi.com/1422-0067/21/3/1154/s1.
