# Supplementary material for: Chronic Mild Stress Modified Epigenetic Mechanisms Leading to Accelerated Senescence and Impaired Cognitive Performance in Mice
Source: Int J Mol Sci. 2020 Feb 10;21(3):1154. doi: 10.3390/ijms21031154 (PMC7037343; doi:10.3390/ijms21031154)

Supplementary Table 1. Antibodies used in Western blot studies.

Antibody	Host	Source/Catalog	WB dilution
**Acetyl H3**	Rabbit	Millipore/06-599	1:1000
**H3**	Rabbit	Cell Signaling/#9715	1:1000
**Acetyl H4**	Sheep	R&D Systems/AF5215	1:1000
**H4**	Rabbit	Cell Signaling/#2592	1:1000
**HDAC2**	Rabbit	Abcam/ab-12169	1:1000
**p-H2A.X S139**	Rabbit	Cell Signaling/#9718	1:1000
**H2A.X**	Rabbit	Cell Signaling/#2595	1:1000
**H3K9me2**	Rabbit	Cell Signaling/#4658	1:1000
**TET2**	Mouse	Santa Cruz/sc-398535	1:500
**β-Catenin**	Goat	Santa Cruz/sc-7963	1:500
**BACE1**	Rabbit	Cell Signaling/#5606	1:2000
**sAPPβ**	Rabbit	Covance/SIG-39139	1:1000
**ADAM10**	Rabbit	Abcam/ab1997	1:1000
**mTORC1**	Rabbit	Cell Signaling/#2501	1:1000
**BCL2**	Mouse	Cell Signaling/#2870	1:1000
**NRF2**	Rabbit	Santa Cruz/sc-722	1:500
**GPX1**	Rabbit	Novus Biological/NBP1-33620	1:1500
**SOD1**	Mouse	Calbiochem/574597	1:1000
**Catalase**	Rabbit	Abcam/ab16731	1:1000
**NF-κβ**	Rabbit	Cell Signaling/DE14E12	1:1000
**p-Tau S396**	Rabbit	Invitrogen/44752G	1:1000
**p-Tau S404**	Rabbit	Invitrogen/44758G	1:1000
**Tau**	Goat	Santa Cruz/sc-1995	1:500
**p-GSK3β S9**	Rabbit	Millipore/Clone 12B2	1:1000
**GSK3β**	Rabbit	Cell Signaling/#9315	1:1000
**Beclin 1**	Rabbit	Abcam/ab62557	1:1000
**p-mTOR S2481**	Rabbit	Santa Cruz/sc-293089	1:500
**mTOR**	Rabbit	Novus Biologicals/NB100-240	1:1000
**LC3B**	Rabbit	Cell Signaling/#2775	1:1000
**GAPDH**	Mouse	Millipore/MAB374	1:2000
**β-Tubulin**	Mouse	Millipore/Clone AA2	1:2000
**Goat-anti-mouse HRP conjugated**		Biorad/170-5047	1:2000
**Goat-anti-rabbit HRP conjugated**		Biorad/170-6515	1:2000
**Donkey-anti-goat HRP conjugated**		Santa Cruz/sc-2020	1:2000

Supplementary Table 2. Primers and probes used in qPCR studies.

SYBR-Green primers

Target	Product size (bp)	Forward primer (5′-3′)	Reverse primer (5′-3′)
Sirt1	229	AACACACACACAAAATCCAGCA	TGCAACCTGCTCCAAGGTAT
Sirt6	147	CGTTAATCGGTATGCCGT	GCAATTAGCGGATAACGG
Dnmt3a	142	TGCCAGACCTTGGAAACCTC	GCTGGCACCCTCTTCTTCAT
Aox1	286	CATAGGCGGCCAGGAACATT	TCCTCGTTCCAGAATGCAGC
Il-6	112	ATCCAGTTGCCTTCTTGGGACTGA	TAAGCCTCCGACTTGTGAAGTGGT
Il-10	105	GGCGCTGTCATCGATTTCT	TGGCCTTGTAGACACCTTG
Tnf-α	157	TCGGGGTGATCGGTCCCCAA	TGGTTTGCTACGACGTGGGCT
Gfap	125	CCTTCTGACACGGATTTGGT	ACATCGAGATCGCCACCTAC
Aβ-precursor	99	TCGGGGTGATCGGTCCCCAA	GTCACGTTCACCCTCCCCAG
β-actin	218	CTGTCCCTGTATGCCTCTG	ATGTCACGCACGATTTCC

miRCURY LNA miRNA Validation probes

Target	Reference
hsa-miR-29c-3p	YP00204729
hsa-miR-431-5p	YP00204737
mmu-miR-298-5p	YP00205092
mmu-miR-98-5p	YP00204640
hsa-miR-140-5p	YP00204540
hsa-miR-181a-5p	YP00206081
hsa-miR-106b-5p	YP00205884
SNORD68	YP00203911

Supplementary Table 3. MicroRNAs differentially expressed in the hippocampus of SAMP8 compared to SAMR1. In bold significant changes in microRNAs gene expression. Chronic mild stress (CMS).

miRNAs	t-test R1vsP8 (Control)	t-test R1vsP8 (CMS)	SAMP8 mean (Control)	SAMP8 mean (CMS)
**mmu-let-7c-5p**	0,5206	0,8132	0,685515425	1,371331756
**mmu-let-7e-5p**	**0,0811**	0,5587	2,40490477	1,632769635
**mmu-miR-106b-5p**	0,9999	0,2660	1,132383859	1,232476548
**mmu-miR-107-3p**	0,8380	0,4660	1,328378691	3,799918898
**mmu-miR-128-3p**	0,4700	0,8359	1,019540454	0,770764343
**mmu-miR-140-5p**	**0,0888**	0,7931	0,427932135	0,638528798
**mmu-miR-146a-5p**	0,3321	0,9831	0,661767151	1,05660245
**mmu-miR-148b-3p**	0,5777	0,7738	0,850640236	1,358667617
**mmu-miR-181a-5p**	0,3322	0,5831	0,648670009	0,807795594
**mmu-miR-191-5p**	0,4023	0,7820	1,49960659	2,283907188
**mmu-miR-26b-5p**	0,9616	0,7750	1,256561233	2,40301896
**mmu-miR-27a-3p**	0,9834	0,4614	1,094208773	1,473142951
**mmu-miR-29a-3p**	0,6635	0,9001	0,781562275	0,780564672
**mmu-miR-29c-3p**	0,7673	0,2989	0,872848654	1,075036978
**mmu-miR-431-5p**	0,2351	0,3631	0,647237869	0,8543147
**mmu-miR-484**	0,4205	0,5736	0,697625273	0,85411048
**mmu-miR-7a-5p**	0,9262	0,1577	0,970688911	0,817409622
**mmu-miR-9-5p**	0,6745	0,7763	0,86533627	1,103131318
**mmu-miR-98-5p**	0,6566	**0,0789**	0,797413303	1,203181993
**mmu-miR-101b-3p**	0,7844	0,8586	0,973139682	1,197227365
**mmu-miR-151-3p**	0,3281	**0,0705**	0,670170619	1,655517937
**mmu-miR-298-5p**	0,5110	0,8686	0,506346238	1,199505517

Supplementary Table 4. MicroRNAs differentially expressed in the hippocampus of SAMP8 and SAMR1 under the influence of Chronic Mild Stress (CMS) compared to Control. In bold significant changes in microRNAs gene expression.

miRNAs	t-test CMSvsCt (SAMR1)	t-test CMSvsCt (SAMP8)	CMS mean (SAMR1)	CMS mean (SAMP8)
**mmu-let-7c-5p**	0,6702	0,4110	1,217779659	1,371331756
**mmu-let-7e-5p**	0,3220	0,2598	2,329973466	1,632769635
**mmu-miR-106b-5p**	0,4796	0,8131	0,674459492	1,232476548
**mmu-miR-107-3p**	0,1868	0,1199	2,889824565	3,799918898
**mmu-miR-128-3p**	0,2969	0,4166	0,721771206	0,770764343
**mmu-miR-140-5p**	**0,0284**	0,6703	0,520726166	0,638528798
**mmu-miR-146a-5p**	0,9188	0,5186	0,661767151	1,05660245
**mmu-miR-148b-3p**	0,9315	0,6381	1,007171102	1,358667617
**mmu-miR-181a-5p**	0,8769	0,6623	1,136036714	0,807795594
**mmu-miR-191-5p**	0,2235	0,5449	1,930735094	2,283907188
**mmu-miR-26b-5p**	0,4720	0,4802	1,945274377	2,40301896
**mmu-miR-27a-3p**	0,7589	0,4660	1,276677933	1,473142951
**mmu-miR-29a-3p**	0,5346	0,9984	0,731789022	0,780564672
**mmu-miR-29c-3p**	0,6103	0,6891	0,821643978	1,075036978
**mmu-miR-431-5p**	0,2273	0,3696	0,670621426	0,8543147
**mmu-miR-484**	0,4220	0,5712	0,705959538	0,85411048
**mmu-miR-7a-5p**	0,2673	0,1856	1,221853394	0,817409622
**mmu-miR-9-5p**	0,8555	0,4140	1,15465838	1,103131318
**mmu-miR-98-5p**	0,5442	0,3852	0,787400729	1,203181993
**mmu-miR-101b-3p**	0,8696	0,6396	1,281426619	1,197227365
**mmu-miR-151-3p**	0,6778	0,4055	1,172720512	1,655517937
**mmu-miR-298-5p**	0,9961	0,2580	1,085941122	1,199505517

Supplementary Figure 1. Representative Western Blot for SIRT1 (A) and SIRT2 (B) protein levels and quantification. Relative gene expression of *Sirt2* (C). Gene expression levels were determined by real-time PCR. Values in bar graphs are adjusted to 100% for protein levels of SAMR1 Control (R1 Ct). Values are mean ± Standard error of the mean (SEM); (n = 4 for each group). *p<0.05.

A

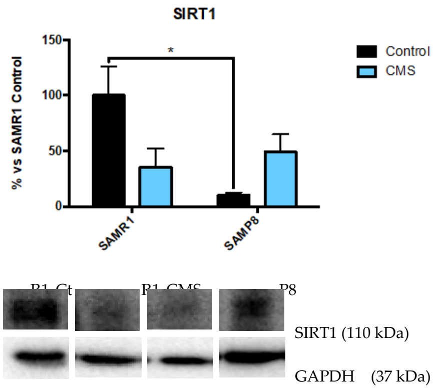

B

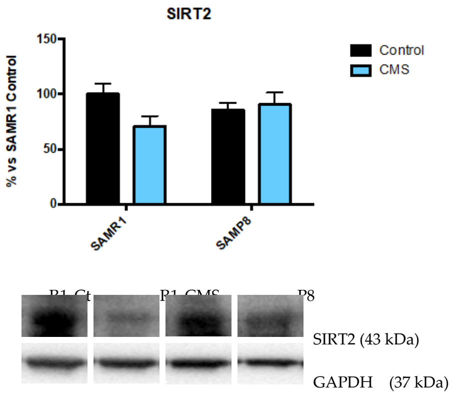

C

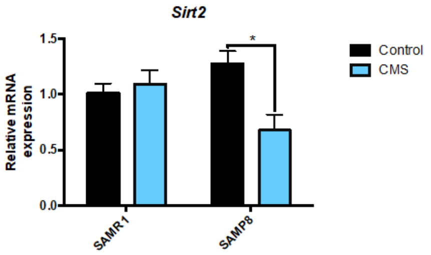

Supplementary Figure 2. Representative changes

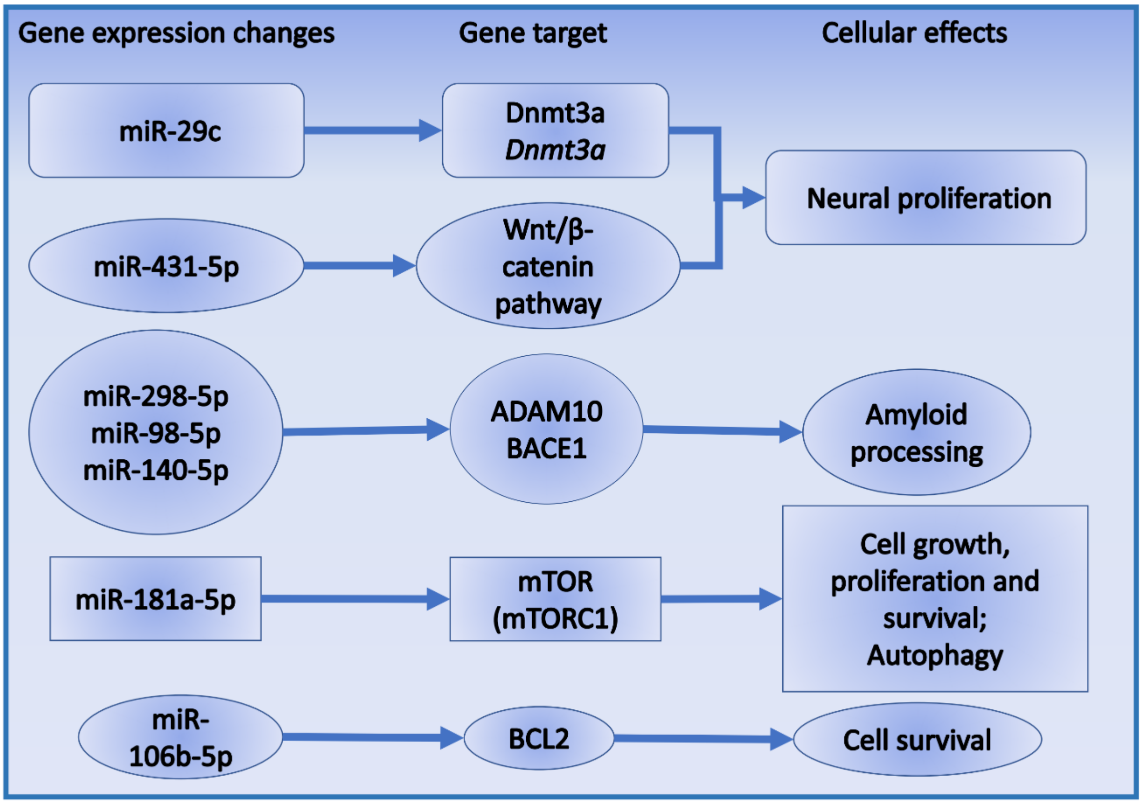

Supplement: Supplementary file 1 [file ijms-21-01154-s001.pdf]
